# Advance in biologics for chronic rhinosinusitis with nasal polyps

**DOI:** 10.3389/falgy.2026.1759649

**Published:** 2026-02-27

**Authors:** Yiming Di, Ruizhi Zeng, Lei Huang, Yong Wu, Qingwu Wu

**Affiliations:** 1 Department of Otorhinolaryngology Head and Neck Surgery, The Third Affiliated Hospital of Sun Yat-sen University, Guangzhou, China; 2 Zhongshan School of Medicine, Sun Yat-sen University, Guangzhou, China

**Keywords:** biologics, chronic rhinosinusitis with nasal polyps, drug switching, efficacy, endoscopic sinus surgery, safety, type 2 inflammation

## Abstract

Managing chronic rhinosinusitis with nasal polyps (CRSwNP) poses challenges, especially when conventional treatments fail to achieve adequate symptom control. CRSwNP is often marked by type 2 inflammation, and a large number of patients have other comorbid type 2 conditions, such as asthma. Currently, 4 biologics have been approved to treat CRSwNP—dupilumab, omalizumab, mepolizumab, and stapokibart (stapokibart is approved in China but not by the US Food and Drug Administration)—with additional promising therapies currently under development. Biologics can enhance the quality of life for CRSwNP patients, reduce the need for systemic corticosteroid therapy and endoscopic sinus surgery (ESS), and improve the management of comorbid conditions. We review clinical trials and real-world data on the effectiveness and safety of biologics for CRSwNP, compare biologic therapy and ESS, and explore the switching and simultaneous use of biologics.

## 1 Introduction

Chronic rhinosinusitis (CRS) encompasses a heterogeneous spectrum of inflammatory disorders affecting the nasal passages, paranasal sinuses, and upper respiratory tract. Symptoms of CRS include nasal congestion or blockage, hyposmia or anosmia, facial pain or pressure, and nasal rhinorrhea (1). CRS is often phenotyped according to the presence (CRSwNP) or absence of nasal polyps. Patients with CRSwNP most frequently experience symptoms, including nasal obstruction and anosmia. It affects 25%–30% of patients with CRS and 1%–4% of the general population, and imposes significant impacts on patients' health-related quality of life (HRQoL), as well as a heavy socioeconomic burden (2, 3). Conventional treatments, such as intranasal corticosteroid (INCS) and endoscopic sinus surgery (ESS), often provide only temporary relief, with frequent recurrence of symptoms. The advent of biologic drugs for type 2 immune effectors such as IL-4, IL-5, and IL-13, as well as IgE, offers new approaches for physicians and hope for patients to manage this difficult disease (4). For patients with severe, refractory CRSwNP, biologic therapies have emerged as a promising treatment option. In this review, we aimed to comprehensively summarize the current advances in biologic therapy for CRSwNP, focusing on biologic efficacy and safety, comparing biologics with ESS, biologic switching between different agents, and the simultaneous use of multiple biologics.

## 2 Efficacy of biologics

Currently, biologics are mainly directed against the following critical inflammatory mediators or their receptors, with their core characteristics summarized in Table 1.

**Table 1:** Summary of biologics’ mechanisms and primary outcomes of pivotal trials.

Drug name	Molecular target	Pivot trials	Primary outcomes (LS mean difference vs. placebo; 95% CI; *p* value)
Dupilumab	IL-4Rα	*SINUS-24* & *SINUS-52*	Change from baseline in NPS and NCS at week 24. *SINUS-24* NPS: −2.06 (−2.43 to −1.69; *P* < 0.0001). NCS: −0.89 (−1.07 to −0.71; *P* < 0.0001). *SINUS-52* NPS: −1.80 (−2.10 to −1.51; *P* < 0.0001). NCS: −0.87 (−1.03 to −0.71; *P* < 0.0001).
Stapokibart	IL-4Rα	*CROWN-2*	Change from baseline in NPS and NCS at week 24. NPS: −2.3 (−2.6 to −1.9; *P* < 0.001). NCS: −0.7 (−0.9 to −0.5; *P* < 0.001).
Omalizumab	IgE	*POLYP-1* & *POLYP-2*	Change from baseline in NPS and NCS at week 24. *POLYP 1* NPS: −1.14 (−1.59 to −0.69; *P* < 0.0001). NCS: −0.55 (−0.84 to −0.25; *P* = 0.0004). *POLYP 2* NPS: −0.59 (−1.05 to −0.12; *P* = 0.014). NCS: −0.50 (−0.80 to −0.19; *P* = 0.0017).
Mepolizumab	IL-5	*SYNAPSE* & *MERIT*	Change from baseline in NPS at week 52 and in mean nasal obstruction VAS score during weeks 49–52. *SYNAPSE* NPS: −0.73 (−1.11 to −0.34 *P* < 0.0001). VAS: −3.14 (−4.09 to −2.18 *P* < 0.0001). *MERIT* (mITT population) NPS: −0.43 (−0.89 to 0.03; *P* = 0.067). VAS: −1.43 (−2.37 to −0.50; *P* = 0.003).
Benralizumab	IL-5R	*OSTRO*	Change from baseline in NPS and patient-reported mean NBS (assessed once every 2 weeks) at Week 40. NPS: −0.570 (−0.852 to −0.289; *P* < 0.001). NBS: −0.270 (−0.458 to −0.083; *P* = 0.005).
Depemokimab	IL-5	*ANCHOR-1* & *ANCHOR-2*	Change from baseline in total NPS at week 52 and mean nasal obstruction VRS score over weeks 49–52. *ANCHOR-1* NPS: −0.7 (−1.1 to −0.3; *P* < 0.001) VRS: −0.23 (−0.46 to 0.00; *P* = 0.047) *ANCHOR-2* NPS: −0.6 (−1.0 to −0.2; *P* = 0.004) VRS: −0.25 (−0.46 to −0.03; *P* = 0.025) Integrated NPS: −0.7 (−0.9 to −0.4; *P* < 0.001*) VRS: −0.24 (−0.39 to −0.08; *P* = 0.003*)
Tezepelumab	TSLP	*WAYPOINT*	Change from baseline in NPS and NCS at week 52. *WAYPOINT* NPS: −2.08 (−2.40 to −1.76; *P* < 0.001). NCS: −1.04 (−1.21 to −0.87; *P* < 0.001).

TSLP, thymic stromal lymphopoietin; NPS, nasal polyp score; NCS, nasal congestion score; VAS, visual analog scale; VRS, verbal response scale; NBS, nasal blockage score.

* Nominally significant *p* value (analysis not included in the hierarchy or adjusted for multiplicity).

### 2.1 Anti-IL-4/IL-13

There are two kinds of biologics, dupilumab and stapokibart, targeting IL-4 Rα, blocking IL-4 and IL-13 signalling. In dupilumab's two phase 3 trials—LIBERTY NP SINUS-24 (24-week follow-up) and LIBERTY NP SINUS-52 (52-week follow-up), dupilumab has been shown to reduce nasal polyp score (NPS), need for systemic corticosteroid (SCS) therapy, and ESS, while also improving nasal congestion score (NCS), the 22-item Sinonasal Outcome Test (SNOT-22), Lund-Mackay computed tomography score, and the University of Pennsylvania Smell Identification Test (UPSIT) score, compared with placebo. Efficacy shown in SINUS-24 diminished after discontinuation while effects shown in SINUS-52 kept improving to week 52, highlighting the need for sustained type 2 inflammation suppression (5). Many real-world studies further validate the efficacy of dupilumab, with the longest follow-up duration reaching 4 years (6). Specifically, a real-world study with a 96-week (2-year) follow-up has shown that dupilumab's efficacy for CRSwNP has been principally established within the first 24 weeks, has persisted through the entire follow-up period, and has yielded only marginal additional improvements thereafter; tapering dupilumab by prolonging the interdose interval—implemented every 24 weeks, conditional on the assessment of biological response and CRS control—can be applied in the vast majority of patients, which may ultimately enhance cost-effectiveness (7).

In the phase 3 clinical trial of stapokibart found that patients with severe CRSwNP treated with a daily intranasal INCS, stapokibart reduced polyp size and severity of nasal symptoms at 24 weeks. CROWNS-2 follow-up duration is 52 weeks. Lack of longer follow-up duration. However, only patients in China were enrolled, limiting generalizability to other populations (8).

### 2.2 Anti-IgE

Omalizumab, an anti-IgE antibody, has demonstrated efficacy in patients with CRSwNP and comorbid asthma previously. In the phase 3 study with a 24-week follow-up duration evaluating the efficacy and safety of omalizumab in patients with severe CRSwNP despite daily INCS therapy, enhancements in NPS and NCS were coincident with important improvements in Total Nasal Symptom Score, SNOT-22 score, sense of smell, postnasal drip, runny nose and UPSIT score (9). After this stage, subjects in both the placebo and omalizumab arms received 28 weeks of open-label omalizumab together with baseline nasal mometasone therapy, and were then monitored for 24 weeks subsequent to the cessation of omalizumab administration. Following omalizumab withdrawal, scores exhibited a gradual decline throughout the 24-week follow-up phase, yet values in both groups still remained superior to the pretreatment baseline (10). Multiple real-world studies of omalizumab in patients with CRSwNP demonstrate similar efficacy to the phase 3 studies (11, 12).

### 2.3 Anti-IL-5

Two monoclonal antibodies, mepolizumab and depemokimab, inactivate IL-5, thereby inhibiting eosinophilic inflammation. In the phase 3 trial SYNAPSE of mepolizumab, which incorporated a 52-week follow-up duration, mepolizumab improved nasal polyp size and nasal obstruction, reduced need for ESS and SCS use, and improved sinonasal symptoms and HRQoL. This trial included only patients with prior surgery, which may be different from other trials (13). The MERIT study of mepolizumab, a 52-week follow-up trial focusing on patients from Japan, China, and Russia with CRSwNP/eosinophilic CRS, has also demonstrated significant improvements in nasal obstruction, sinonasal symptoms, and HRQoL. There are differences in disease profiles between populations in Asia and Europe, and this trial demonstrates efficacy and safety in patients with CRSwNP in these countries (14).

Depemokimab is the first ultra-long-acting anti-IL-5 biological drug allowing biannual administration and sustained suppression of Type 2 inflammation. In the phase 3 ANCHOR-1 and ANCHOR-2 trials, which incorporated a 52-week follow-up duration, participants on depemokimab had significantly greater improvements in the coprimary endpoints of total NPS and participant-reported mean nasal obstruction verbal response scale score, versus placebo. Secondary endpoints also favoured depemokimab, all with nominal significance in pooled analysis (15).

Benralizumab targets the α subunit of the interleukin-5 receptor (IL-5Rα). This process leads to the swift, nearly total depletion of blood eosinophils and a decrease in basophil counts. In the phase 3 OSTRO study—which included 48 weeks of benralizumab administration and a total 80-week follow-up period (with 32 weeks of extended post-treatment monitoring)—benralizumab led to a significant reduction in NPS and nasal blockage compared with placebo, with efficacy sustained even during the post-discontinuation follow-up phase. Additionally, this study enrolled patients with a total SNOT-22 score ≥ 30, which may lead to an underestimation of the actual effectiveness of these treatments (16, 17).

### 2.4 Anti-TSLP

Tezepelumab is a human monoclonal antibody that specifically prevents thymic stromal lymphopoietin (TSLP) from engaging with its heterodimeric receptor. It is approved for maintenance therapy of severe asthma, regardless of the T2 endotype. In the phase 3 trial of tezepelumab for severe CRSwNP (WAYPOINT), which featured a 52-week follow-up duration, tezepelumab therapy led to significantly greater reductions in the size of nasal polyps, the severity of nasal congestion and sinonasal symptoms, and the use of ESS and SCS than placebo in adults with severe, uncontrolled chronic rhinosinusitis with nasal polyps (18).

### 2.5 Which is the best biologic

Several meta-analyses of randomized controlled trials (RCTs) have indirectly compared the biologics, consistently concluding that dupilumab has superior efficacy (19–23). Notably, in a recent multicentre, randomised, double-blind, phase 4 trial comparing efficacy and safety of dupilumab vs. omalizumab in patients with severe CRSwNP and comorbid asthma, a head-to-head comparison was conducted. Dupilumab showed superiority over omalizumab in meeting the primary endpoints (NPS and UPSIT) and demonstrating significant improvement in all secondary endpoints, including asthma endpoints (24). Further research is warranted to substantiate this conclusion.

### 2.6 Biologics under clinical trials

A marked increase has been seen in clinical trials assessing the safety and efficacy of novel biologics for the treatment of CRSwNP. As summarized in Table 2, numerous late-phase clinical trials are currently investigating biologics targeting upstream epithelial cytokines such as IL-33 and TSLP. Compared with conventional type 2 cytokine blockade, these emerging therapies may exert broader immunomodulatory effects, representing a potential paradigm shift in the future management of CRSwNP.

**Table 2:** Studies listed in clinicaltrials.gov for biologics in the treatment of CRSwNP.

Investigational medicinal products	Mechanism	Phase	Registration number	Status
Itepekimab	Anti-IL-33	3	NCT06834360	Recruiting
Itepekimab	Anti-IL-33	3	NCT06834347	Recruiting
Etokimab	Anti-IL-33	2	NCT03614923	Completed
TQC2731	Anti-TSLP	2	NCT06036927	Completed
TQC2731	Anti-TSLP	2	NCT06451640	Completed
TQC2731	Anti-TSLP	3	NCT07107256	Recruiting
TQH2722	Anti-TSLP	2	NCT06089278	Completed
TQH2722	Anti-TSLP	2	NCT06439381	Unknown status
Lebrikizumab	Anti-IL-13	3	NCT06338995	Recruiting

These alarmins exert effects on both the innate and adaptive immune systems, thereby holding therapeutic potential for both T2-high and T2-low CRSwNP subtypes. Furthermore, while currently approved biologics provide substantial clinical benefits, their associated economic burden may limit widespread clinical adoption. The development of these novel biologics is expected to provide patients with more cost-effective therapeutic alternatives.

## 3 Adverse events (AEs) of biologics

Based on comprehensive evidence from RCTs, meta-analyses, and other studies, biologics demonstrate significant efficacy in treating CRSwNP, notably reducing the risk of disease progression events such as asthma exacerbation and worsening nasal polyps (5, 25). While their overall tolerability is favorable, vigilance regarding treatment-related AEs remains essential. The most frequently reported AEs, including nasopharyngitis, injection-site reactions, headache, and upper respiratory tract infections (5, 9, 13, 16, 18, 26), are generally mild to moderate, non-specific, and self-limiting (27). Their incidence is comparable to that of placebo, with no reported treatment-related fatal serious AEs. However, each biologic exhibits a distinct safety profile linked to its specific mechanism. Dupilumab is associated with ocular (e.g., conjunctivitis, dry eye) and dermatologic AEs, and is uniquely strongly linked to injection-site reactions (28, 29). Omalizumab requires heightened attention to allergic reaction and pregnancy-related risks (28, 29). Tezepelumab requires monitoring for musculoskeletal and potential cardiac risks (28). Mechanism-related effects, such as hypereosinophilia induced by dupilumab (30) or sustained blood eosinophil count reduction by depemokimab (15), require clinical vigilance. Additionally, biologics including dupilumab, mepolizumab, and omalizumab may more than double the risk of rheumatic AEs (e.g., arthralgia) (31). Therefore, an individualized strategy is imperative—integrating patient comorbidities, tolerance, and drug-specific risks—to implement targeted prevention and monitoring plans, thereby maximizing therapeutic benefits while minimizing risks.

## 4 Biologics vs. ESS

Current treatment guidelines recommend surgical intervention when conventional medical therapy proves ineffective in managing CRSwNP. Both ESS and biologics have demonstrated efficacy in treating patients with CRSwNP who are refractory to conventional medical treatment or have contraindications to such therapy. Comparative analysis of ESS and biologics has thus emerged as a topic of considerable interest.

Biologic therapy has advantages over ESS in the following aspects. Existing research results indicate that in terms of the improvement of nasal congestion symptoms (assessed by NCS), the dupilumab treatment regimen may demonstrate more significant clinical benefits compared with ESS (32). For the evaluation of olfactory function (using the UPSIT-40), patients treated with dupilumab were reported to have significantly greater self-reported olfactory improvement than those treated with ESS (32, 33). Furthermore, in terms of symptom relief, according to SNOT-22, patients treated with dupilumab showed more significant improvements in symptoms such as cough and postnasal drip compared with those in the ESS group (33). The dupilumab group showed greater improvement in Visual Analog Scale (VAS) rhinorrhea/olfaction scores and Smell Identification Test scores. It also demonstrated superior local inflammation control while the requirement for SCS usage was significantly reduced (34). This further supports the potential clinical advantages of dupilumab in alleviating multi-dimensional symptoms in patients with CRSwNP.

Surgery is superior to biologic therapy in the following aspects. Compared with omalizumab, dupilumab, and mepolizumab therapies, ESS shows better clinical efficacy in significantly reducing nasal polyp size (32, 33, 35). Although dupilumab may have similar clinical efficacy to ESS, ESS has been proven to be a more cost-effective treatment strategy based on health economic evaluations. For patients with refractory CRSwNP, ESS should continue to serve as the standard treatment (36). Existing research data indicate that there are no statistically significant differences in safety indicators between ESS and biologics. However, due to the relatively short clinical application time of biologics, their long-term safety profiles still need to be further verified through larger-sample prospective studies.

Based on the evaluation using SNOT-22 scores, both biologics and ESS can significantly improve the symptom burden of patients with CRSwNP, but there is controversy regarding which is superior. According to a meta-analysis, SNOT-22 score of the dupilumab group was inferior to that of the ESS group until 6 months posttreatment, but the scores were similar at around 1 year (32). In addition, the therapeutic advantage of ESS in improving SNOT-22 scores compared with omalizumab is also controversial. Some studies have shown that ESS has a significant therapeutic advantage in SNOT-22 scores (35); however, other studies have indicated that the average improvement magnitude of the SNOT-22 with omalizumab treatment is greater than that with ESS (37).

ESS demonstrates comparable efficacy to biologics such as omalizumab, mepolizumab, and benralizumab in improving patients' HRQoL and symptoms. Studies further indicate that biologics can serve as an alternative or adjuvant treatment for refractory severe CRSwNP (38). For patients with high polyp burden, ESS combined with mepolizumab treatment achieves more significant improvements in HRQoL (SNOT-22 score), NPS, NCS, and CRS Symptom VAS compared to biologic monotherapy (37). Studies have confirmed that both ESS and dupilumab can significantly reduce symptom burden and improve HRQoL in patients with CRS. ESS provides rapid relief from nasal obstruction and may reduce the colonization rate of nasal bacterial biofilms, yet its effect on improving olfactory function is limited. This indicates the complementary value of the two treatment modalities: dupilumab exerts therapeutic effects by targeting and inhibiting the Th2-type inflammatory pathway, while ESS optimizes local medication delivery by correcting anatomical abnormalities (39). Compared with patients receiving omalizumab monotherapy or ESS alone, those with prior ESS treated with omalizumab demonstrated earlier and more pronounced improvements in HRQoL, symptom scores, and endoscopic findings (including NPS and bilateral modified Lund-Kennedy score) as early as Week 16, and these improvements were sustained for 2 years (37).

The current study has the following main limitations: First, due to the relatively recent approval of novel biological agents such as stapokibart, the systematic evaluation of their clinical efficacy and safety remains inadequate, with limited data from direct comparative studies with ESS; Second, existing meta-analyses have insufficient sample sizes of the included studies, which may compromise the robustness of the conclusions. Therefore, multicenter, large-sample RCTs are urgently needed for further validation.

## 5 Switching biologics in CRSwNP

Although biologics have been employed to treat CRSwNP, not all patients respond favorably, necessitating the use of other biologics. Given the current lack of relevant clinical guidelines, the following paragraphs focus on examining the specific process and underlying rationale for switching biologic therapies in the treatment of CRSwNP.

### 5.1 Reasons for switching biologics

#### 5.1.1 Lack of efficacy

For patients with CRSwNP, one of the most frequent rationales for switching to alternative biologic agents is either insufficient therapeutic efficacy or a partial clinical response to the initial biologic treatment. Definitions of inadequate symptom control during biologic treatment vary across real-world studies, typically encompassing the following key aspects: severe CRS symptoms (including anosmia), increased NPS, persistent requirement for SCS, and the need for surgical intervention. Lack of efficacy was the main reason for switching off mepolizumab and omalizumab (40). Brkic et al. studied the efficacy and safety of switching biologics in patients with severe CRSwNP and non-steroidal anti-inflammatory drug-exacerbated respiratory disease. The main type of biologic switching is from omalizumab to dupilumab. Among the reasons for switching, the most common is worsening nasal congestion, accounting for 70.6% of patients, followed by deterioration of olfactory function (anosmia/hyposmia) in 23.5% of patients, while only 5.9% of switched patients did so due to the AE of joint pain (41).

#### 5.1.2 The length of therapy

The length of therapy is another important factor. The European position paper and consensus on CRSwNP recommend evaluations at 4–6 months and re-evaluations at 1 year (2, 4, 42). Chinese position paper unanimously advocates for a more frequent follow-up schedule. After 3 months of treatment, if the reduction in NPS fails to reach ≥1 point and the patient is unsatisfied with their symptom relief, discontinuing the current agent or switching to an alternative biologic therapy is needed; if treatment is continued beyond the 3-month mark but the NPS reduction still does not meet the ≥1-point threshold at the 6-month evaluation, this will also prompt the decision to discontinue or switch the biologic agent (17). Although biologic agents are usually well-tolerated, they are not exempt from AEs. Although most of the AEs are mild and transient, they will still bother some patients and prompt them to seek alternatives. Rare but serious AEs also drive such switching decisions. Dorling et al. observed that AEs were the leading cause for switching off dupilumab (40).

#### 5.1.3 Comorbidities

Comorbid conditions may prompt the switching of biologics to more effectively manage not only CRSwNP symptoms but also the accompanying disorders. Most of the existing studies also focus on comorbidities like asthma. Initial selection of biologics can be guided by the predominant disease or key symptoms. Nevertheless, the therapeutic effects of biologics may adequately control CRSwNP yet fail to provide sufficient management of asthma, vice versa (40, 43). Therefore, adjusting biologics until both CRSwNP and asthma symptoms are adequately controlled represents a reasonable therapeutic strategy. Patients with CRSwNP comorbid with asthma may thus benefit from switching to dupilumab when other biologics fail to achieve optimal symptom management (17).

### 5.2 Criteria for switching biologics

Studies have repeatedly emphasized that the core principle of biologic therapy for CRSwNP lies in targeting the dominant inflammatory pathway: although asthma is well controlled, biologics targeting IL-5 or IgE may on occasion fail to achieve disease remission in CRSwNP. In such cases, switching to biologics that target the IL-4/IL-13 pathway may offer superior efficacy (40, 41, 43). Significant differences exist in the application scenarios and therapeutic characteristics of different biologics, as detailed below:

Dupilumab: This drug exhibits a faster rate of symptom relief than other biologics and serves as the preferred treatment option for cases of urgency (44). It acts on the more upstream IL-4 pathway in the inflammatory cascade, thereby offering distinct advantages for patients with multisystem atopic diseases or those with CRSwNP comorbid with asthma (40). It is capable of simultaneously ameliorating lesions in both the upper and lower airways, thereby achieving dual therapeutic benefits (43–45). For patients who failed initial anti-IL-5 therapy, approximately 50% can achieve effective disease control with dupilumab (43).

Anti-IL-5 agents: It is important to note that if initial anti-IL-5 therapy fails, switching to a second anti-IL-5 agent generally fails to improve patient outcomes (43).

Omalizumab: This agent targets the IgE pathway and is more suitable for CRSwNP patients with comorbid severe allergies, exerting faster and more significant effects in alleviating allergy-related symptoms (46).

In addition, the following key considerations should be integrated into clinical decision-making: clarifying the causes of previous biologic treatment failure is of paramount importance; for patients with multiple comorbid type 2 inflammation-related diseases, priority should be accorded to agents that alleviate the core symptoms most distressing to the patient (40, 41); efficacy assessment must simultaneously address the control of upper and lower airway symptoms, and if a single biologic fails to achieve comprehensive coverage, simultaneous biologic use may be considered (43).

### 5.3 Multiple switches and simultaneous biologic using

If patients exhibit an incomplete response to biologic therapy or intolerance to AEs, multiple adjustments to the biologic regimen may be necessary. Although the clinical application of biologic combination therapy remains limited, it is suitable for complex cases with inadequate response to monotherapy—particularly in scenarios involving overlapping disease mechanisms, such as severe asthma combined with CRSwNP. For example, the combination of omalizumab and dupilumab can yield additive therapeutic benefits in some refractory cases. The research team reported that, based on their experience to date, this approach could be implemented safely but requires close clinical monitoring (43). In such complex scenarios, individualized treatment plans should be developed, and multidisciplinary decision-making should be adopted to optimize treatment outcomes. Both multiple regimen adjustments and biologic combination therapy are specialized treatment strategies and are only applicable to patients in whom monotherapy fails to achieve disease control (40).

### 5.4 Transitioning process between biologics

There is currently no clear clinical consensus on whether a washout period is required when switching biologics for the treatment of CRSwNP. Evidence from studies of switching without a washout period has shown that this approach enables rapid symptom improvement and sustained disease control after switching, without increasing the risk of AEs, and is associated with a lower risk of treatment discontinuation and subsequent symptom exacerbation (41, 47). In contrast, studies involving a washout period indicate that symptom control temporarily declines during the washout phase, which may negatively impact HRQoL, and this approach does not demonstrate a clear safety advantage (48). This may be related to the following mechanism: when patients switch from anti-IL-5 therapy to dupilumab, it is recommended to initiate dupilumab directly and leverage the low eosinophil levels induced by prior anti-IL-5 treatment to minimize the risk of eosinophilia-related complications (43). Therefore, provided that there are no contraindications or particular safety considerations, switching biologics without a washout period may serve as a feasible and efficacious strategy for the management of CRSwNP.

## 6 Conclusion

Biologics targeting different molecules in type 2 inflammation have brought new opportunities for the treatment of CRSwNP, but attention should be paid to their specific AEs. Both biologics and ESS have their own advantages and disadvantages and are complementary and synergistic: the former target and inhibit inflammatory pathways to improve multidimensional symptoms, while the latter correct anatomical abnormalities and rapidly reduce the size of nasal polyps. Patients with CRSwNP often need to switch biologics due to insufficient efficacy, AEs, failure to meet treatment evaluation criteria, or the need to manage comorbidities. Dupilumab is particularly suitable for patients unresponsive to other agents. In the absence of contraindications, switching biologics without a washout period is a feasible strategy balancing efficacy and safety. Clinically, individualized treatment plans should be developed based on patients' individual conditions.
